# Annexins Bridging the Gap: Novel Roles in Membrane Contact Site Formation

**DOI:** 10.3389/fcell.2021.797949

**Published:** 2022-01-06

**Authors:** Carlos Enrich, Albert Lu, Francesc Tebar, Carles Rentero, Thomas Grewal

**Affiliations:** ^1^ Departament de Biomedicina, Unitat de Biologia Cel·lular, Facultat de Medicina i Ciències de la Salut, Universitat de Barcelona, Barcelona, Spain; ^2^ Centre de Recerca Biomèdica CELLEX, Institut d’Investigacions Biomèdiques August Pi i Sunyer (IDIBAPS), Barcelona, Spain; ^3^ School of Pharmacy, Faculty of Medicine and Health, University of Sydney, Sydney, NSW, Australia

**Keywords:** annexins, membrane contact sites, endolysosomes, mitochondria, endoplasmic reticulum, cholesterol, calcium-binding proteins, lipid transport

## Abstract

Membrane contact sites (MCS) are specialized small areas of close apposition between two different organelles that have led researchers to reconsider the dogma of intercellular communication *via* vesicular trafficking. The latter is now being challenged by the discovery of lipid and ion transfer across MCS connecting adjacent organelles. These findings gave rise to a new concept that implicates cell compartments not to function as individual and isolated entities, but as a dynamic and regulated ensemble facilitating the trafficking of lipids, including cholesterol, and ions. Hence, MCS are now envisaged as metabolic platforms, crucial for cellular homeostasis. In this context, well-known as well as novel proteins were ascribed functions such as tethers, transporters, and scaffolds in MCS, or transient MCS companions with yet unknown functions. Intriguingly, we and others uncovered metabolic alterations in cell-based disease models that perturbed MCS size and numbers between coupled organelles such as endolysosomes, the endoplasmic reticulum, mitochondria, or lipid droplets. On the other hand, overexpression or deficiency of certain proteins in this narrow 10–30 nm membrane contact zone can enable MCS formation to either rescue compromised MCS function, or in certain disease settings trigger undesired metabolite transport. In this “*Mini Review*” we summarize recent findings regarding a subset of annexins and discuss their multiple roles to regulate MCS dynamics and functioning. Their contribution to novel pathways related to MCS biology will provide new insights relevant for a number of human diseases and offer opportunities to design innovative treatments in the future.

## Introduction

Despite the identification of membrane contacts between neighbouring organelles in the early times of electron microscopy, these small microdomains only received greater attention in the last decade. Two findings prompted further research on the structure and function of membrane contact sites (MCS). Firstly, the endoplasmic reticulum (ER) representing a dynamic 3D network of cisternae and tubules, it fills the cytoplasm and is in physical contact with other organelles (Nixon-Abell et al., 2016). Secondly, the discovery of the physiological relevance of contacts between the ER and mitochondria (mitochondria-associated membranes, MAMs) in the synthesis and exchange of lipids and calcium (Ca^2+^) homeostasis (Vance, 1990). Since then, the MCS-related Universe expanded rapidly and MCS are now considered metabolic platforms for the transport of small molecules such as lipids and ions (Prinz et al., 2020). In addition, MCS modulate various other functions, including organelle trafficking, endosome maturation and positioning, membrane dynamics, Ca^2+^ signalling, autophagy and membrane/vesicle/organelle fusion and fission (Friedman et al., 2013; van der Kant and Neefjes, 2014; Raiborg et al., 2015a; Raiborg et al., 2015b; Eisenberg-Bord et al., 2016; Abrisch et al., 2020; Silva et al., 2020). Membrane contacts also exist in organelles with internal membranes, including mitochondria, chloroplasts, and multivesicular bodies (MVBs) (Prinz et al., 2020). As MCS formation can be manipulated experimentally and is de-regulated in human disease models (Wu et al., 2018; Henne, 2019; Scorrano et al., 2019; Ballabio and Bonifacino, 2020; Martello et al., 2020; Petkovic et al., 2021), MCS have become attractive therapeutic targets.

MCS appear as small contact zones between neighbouring organelles, but contain a plethora of proteins and lipids. The biogenesis, maintenance, dynamics, and function of MCS rely on protein-protein interactions bridging apposed membranes to establish their communication. These proteins in MCS include numerous lipid and cholesterol transfer proteins, and a variety of tethers, sorting nexins, membrane channels, SNAREs, small Rab-GTPases and their regulators. In addition, as outlined in more detail below, several annexins are also found in MCS. It would go beyond the scope of this *Mini Review* to list all proteins and we recommend excellent articles and reviews on this topic (Alpy and Tomasetto, 2005; Eisenberg-Bord et al., 2016; Kentala et al., 2016; Atakpa et al., 2018; Hoyer et al., 2018; Kumar et al., 2018; Quon et al., 2018; Sandhu et al., 2018; Balla et al., 2019; Bohnert, 2019; Liao et al., 2019; Patel, 2019; Cremer et al., 2020; Islinger et al., 2020; Meneses-Salas et al., 2020b; Peng et al., 2020; Hewlett et al., 2021; Reinisch and Prinz, 2021; Saric et al., 2021; Wu and Voeltz, 2021).

### Annexins—Novel Players in Intracellular Communication and Membrane Contact Sites

**FIGURE 1:**
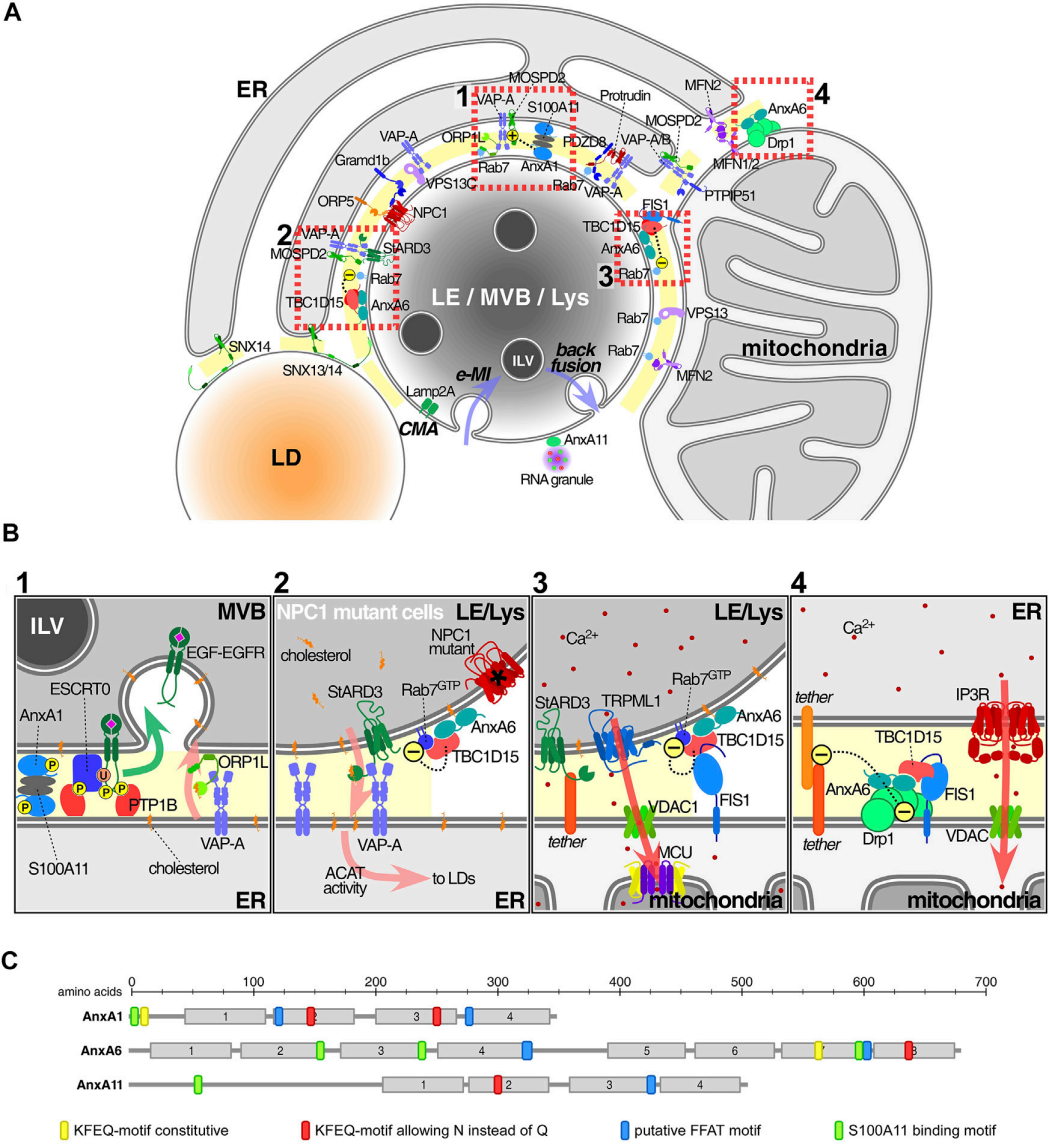
Annexins and associated protein complexes at membrane contact sites. **(A)** Endolysosomes (LE/MVBs/Lys) establish multiple membrane contact sites (MCS) with a variety of other organelles, in particular the ER and mitochondria, but also peroxisomes and lipid droplets. Here we only illustrate a subset of contacts and contact site proteins. Lipid transfer, including cholesterol as well as Ca^2+^ mobilization and signalling are probably the most important MCS-associated functions. Annexins have been found in MCS connecting the ER with either LE or mitochondria (MAMs), as well as in LE-mitochondria contacts. Other proteins or protein complexes serve as tethers to ensure the formation of MCS, including several sorting nexins (Henne et al., 2015; Dong et al., 2016; Saric et al., 2021), VAP proteins, PTPIP51, PDZD8 or protrudin (Shirane et al., 2020; Neefjes and Cabukusta, 2021). Furthermore, ORP5/8 and NPC1/Gramd1 also translocate lipids including PS and cholesterol, across MCS between the ER and mitochondria and/or LE respectively (Galmes et al., 2016; Hoglinger et al., 2019). At the bottom of this endolysosome, we contemplate the possible recruitment of cytosolic annexins into ILV (see text for details) *via* two autophagic pathways. While chaperone-mediated and Lamp2a-dependent autophagy (CMA) may carry annexins into the lumen of MVBs or the outer membrane of ILVs, endosomal microautophagy (e-MI) may board annexins inside ILV (both routes *via* KFERQ-motif) (Tekirdag and Cuervo, 2018). In any case, whatever the topology, these proteins have to escape from lysosomal degradation (Meneses-Salas et al., 2020b). Then, ILV might undergo back fusion/retrofusion (Eden and Futter, 2021), delivering annexins and/or other cargo out of the MVB into the cytosol in the vicinity of MCS, where a suitable local Ca^2+^ and lipid microenvironment could then favour retention in MCS. Alternatively, MVB diversion to exocytosis could generate exosomes. This could be the destination of AnxA11, highly enriched in exosomes. Otherwise, cytosolic AnxA11 could be confined to PI(3,5)P2 at the endolysosomal cytosolic membrane to tether RNA granules (Liao et al., 2019). **(B) 1**: AnxA1, together with S100-A11 as a tetrameric complex, tethers MCS to mediate cholesterol transport from ER to LE/Lys *via* interaction of VAP-A with ORP1L (Eden et al., 2016). In this scenario, AnxA1 overexpression increases MCS between MVB and ER (Wong L. H. et al., 2018). **2**: AnxA6 overexpression decreased MCS numbers between ER and LE/Lys, whereas AnxA6 depletion in NPC1 mutant cells stimulated MCS formation. The underlying mechanism involves the recruitment of AnxA6 and TBC1D15 to Rab7-positive organelles and was associated with increased LE motility and LE-cholesterol release into the ER, through StARD3-VAP-A in MCS (Meneses-Salas et al., 2020b). **3**: TBC1D15 and Rab7 in complex with FIS1 between LE/Lys and mitochondria affects the fission of mitochondria (Wong Y. C. et al., 2018). **4**: AnxA6 interacts with Drp1 and FIS1 between ER and mitochondria to modulate Ca^2+^ dynamics and mitochondrial fission (Chlystun et al., 2013). In all settings shown in Insets 2-4 the presence of AnxA6 seems to cause MCS untethering. Arrows indicate the following: translocation of EGFR-EGF into ILV (green, inset 1), cholesterol flux (pink, insets 1-2), Ca^2+^ flux (red, insets 3-4). **(C)** Schematic representation of the domain structure of the three annexins found in MCS: Motifs that may be involved in the recruitment annexins to MCS are indicated and include the homology to FFAT motifs (blue), S100-binding sites (green) (Rety et al., 2000; Chang et al., 2007; Rintala-Dempsey et al., 2008) and KFERQ-motifs (yellow and red) (Cuervo et al., 2000). Abbreviations that do not appear in the text: ACAT, Acyl-CoA:cholesterol acyltransferase; MCU, mitochondrial calcium uniporter; FIS1, mitochondrial fission 1; IP3R, inositol 1,4,5-triphosphate receptor; TRPML1, transient receptor potential mucolipin 1; Vps13, Vacuolar protein sorting-associated protein 13; MFN1/2, mitofusin1/2; Gramd1b, GRAM domain containing 1B; VAP proteins, VAP-A, VAP-B; monomer specific d-peptide 1 (MOSD1, 2 and 3) and PS, phosphatidylserine.

Annexins are characterized by a high structural homology that enables binding to acidic phospholipids in a Ca^2+^-dependent manner. In fact, most annexin functions in cells are due to their dynamic and reversible binding to membranes (Gerke et al., 2005; Enrich et al., 2017). More recently, mouse models lacking individual annexins validated proposed functions in complex physiological processes *in vivo* (Alvarez-Guaita et al., 2020; Grewal et al., 2021). Annexins are present in multiple cellular compartments to regulate numerous functions, including membrane trafficking, cytoskeleton dynamics, ion channels, cell signalling, membrane repair and pro- or anti-inflammatory activities. Most relevant for this review, annexins (AnxA1, A6 and A11) are located in endolysosomes in the vicinity of the ER (Figure 1A) with novel MCS-related roles for these annexins in cell physiology (Pons et al., 2000; Eden et al., 2016; Liao et al., 2019).

AnxA1 is ubiquitously expressed and found at the plasma membrane, endo-/exocytic vesicles, the cytoskeleton and nucleus (Grewal et al., 2021). Moreover, AnxA1 acts as a tether connecting the ER and MVBs (Eden et al., 2010; Eden et al., 2016). Tyrosine phosphorylated AnxA1 together with its binding partner S100A11 provide a docking site for tyrosine phosphatase 1B (PTP1B), an enzyme localized in the ER, at MVB-ER contacts to enable sorting of ligand-stimulated epidermal growth factor receptor (EGFR) onto intraluminal vesicles (ILVs) (Figure 1B1). EGF-induced AnxA1 phosphorylation is crucial for the segregation of EGFR onto ILVs (White et al., 2006). It remains to be clarified if AnxA1 acts in concert with the ESCRT complex and associated proteins and lipids (Raiborg and Stenmark, 2009) to facilitate this indispensable step targeting EGFR to lysosomes for degradation. Thus, MVB-ER contacts may provide localized sites where the phosphorylation status of AnxA1 and MVB sorting machinery components could be tightly controlled (Futter et al., 1993; White et al., 2006; Du et al., 2012; Eden et al., 2016).

Importantly, the AnxA1 tethering function is also required for ER-derived cholesterol transport to MVBs, a critical step for ILV formation to spatially regulate EGFR signalling. When cholesterol levels are low due to reduced endocytosis of Low-density lipoproteins (LDL), AnxA1-regulated membrane contacts facilitate cholesterol transfer from the ER to MVBs *via* the interaction of ER-localized VAMP-associated proteins (VAPs) and the endosomal oxysterol-binding protein related protein 1L (ORP1L) (Eden et al., 2016).

Besides the AnxA1-S100A11 complex, other annexins also interact with S100 proteins, and may interact with two membranes simultaneously (Rescher and Gerke, 2004; Rintala-Dempsey et al., 2008). Hence, the reversible membrane binding capacity of annexins could establish initial protein-protein (or protein-phospholipid) interactions between LE/MVB and ER membranes to induce MCS formation and allow the exchange of ions and lipids, including cholesterol in other physiological settings (Miwa et al., 2008; Rintala-Dempsey et al., 2008).

AnxA6 has recently also been associated with MCS formation (Meneses-Salas et al., 2020b). Further to its association with the plasma membrane, endo-/exocytic vesicles, mitochondria and lipid droplets (Grewal et al., 2021), AnxA6 was additionally detected in MAMs (Sala-Vila et al., 2016), specialized membrane subdomains enriched in cholesterol and neutral lipids that permit the communication between the ER and mitochondria (Vance, 1990; Hayashi and Su, 2003).

Given the recently identified role of AnxA6 in cholesterol transfer across MCS (Meneses-Salas et al., 2020b) and its presence in MAMs (Sala-Vila et al., 2016), the control of cholesterol transfer across MAMs and its alignment with steroid, oxysterol and bile acid synthesis is decisive for proper mitochondrial homeostasis. Emerging molecular insights include the identification of the founder member of the START [(Steroidogenic Acute Regulatory protein) related lipid transfer] domain family, StAR, also known as StARD1, at the outer mitochondrial membrane (OMM) as part of a multi-protein complex, with the voltage-dependent anion-selective channel protein (VDAC) and phosphate carrier protein (PCP), involved in the import of cholesterol (Reitz et al., 2008; Alpy and Tomasetto, 2014; Elustondo et al., 2017). StARD1 first incorporates ER-derived cholesterol into OMM, and together with VDAC and the translocator protein (TSPO), interacts with ATPase family AAA domain containing 3A (ATAD3A) and cytochrome P450 family 11 subfamily A member 1 (CYP11A1) in the inner mitochondrial membrane (Rone et al., 2012), to move and then metabolize cholesterol (Elustondo et al., 2017). Yet, how the feedback loop that coordinates LDL-derived cholesterol uptake and *de novo* cholesterol synthesis links to tethering and untethering events between the ER or LE/Lys with mitochondria to prevent excessive cholesterol transfer across MAMs remains unclear. Despite regulatory roles for AnxA6 in cholesterol transfer across LE/Lys-ER contacts (see below), similar functions for AnxA6 in MAMs have yet to be identified.

This indicates that mechanisms are in place that keep alternative and NPC1-independent cholesterol transport in an inactive state and do not enable other transport machinery to overcome cholesterol accumulation caused by NPC1 deficiency. Therefore, the presence of yet unidentified inhibitory proteins that act as “gatekeepers” may control activation of alternative cholesterol transport routes exiting LE/Lys. Indeed, NPC1 deficiency was associated with downregulation of the GTPase Rab7, the master regulator of LE/Lys function. Inhibition of Rab7 activity was mediated by AnxA6, which recruited the Rab7-GTPase activating protein (Rab7-GAP) TBC1 domain family member 15 (TBC1D15) to cholesterol-rich LE, thereby lowering Rab7-GTP levels. Strikingly, AnxA6 depletion in NPC1 mutant cells and the concomitant loss of TBC1D15 membrane targeting elevated Rab7-GTP levels, leading to increased MCS formation between LE/Lys and the ER (Figure 1B2). This MCS restoration enabled cholesterol transfer across LE/Lys-ER contacts *via* the cholesterol transporter StARD3 for storage in lipid droplets. Hence, the AnxA6/TBC1D15 complex could become a potential therapeutic target to slow down the progressive neurodegeneration in NPC disease (Enrich et al., 2019; Meneses-Salas et al., 2020b).

Interestingly, loss of TBC1D15-mediated Rab7-GTP hydrolysis also inhibited the untethering of mitochondria-LE/Lys contacts, disrupting mitochondrial distribution and function in models mimicking Parkinson’s disease pathophysiology (Wong Y. C. et al., 2018; Kim et al., 2021) (Figure 1B3). Similarly, Rab7 mutations with reduced GTPase activity in Charcot-Marie-Tooth type 2B (CMT2B) are linked to defective mitochondria-lysosome contact dynamics (Bucci and De Luca, 2012; Wong et al., 2019). Of note, in mitochondria, AnxA6 also interacts with dynamin-related protein 1 (Drp1) (Chlystun et al., 2013), a GTPase interconnected with Rab7-dependent mitochondrial-LE/Lys contact formation in CMT2B (Wong et al., 2019). Hence, one can envisage a scaffolding role for AnxA6 in Rab7/TBC1D15 and Drp1-dependent dynamics of mitochondria-LE/Lys contacts in these neurological diseases (Figure 1B4).

Studies described above suggest annexin levels to differentially impact on MCS numbers, composition and function. Indeed, AnxA1 depletion markedly reduced MCS connecting EGFR-containing MVBs and the ER, while MCS between EGFR-deficient MVBs and the ER remained unaffected (Eden et al., 2016). Likewise, MCS exist in NPC mutant cells, in particular between LE/Lys and mitochondria (Hoglinger et al., 2019), yet AnxA6 depletion and consequently, loss of TBC1D15 recruitment to LE/Lys in these cells, increased MCS numbers between LE/Lys and the ER for cholesterol transfer, requiring Rab7, and the cholesterol transporter StARD3. Hence, high/low annexin levels acting as tethers (AnxA1) or gatekeepers (AnxA6) will differentially influence MCS protein composition and functions, with consequences for cholesterol transport between organelles. This may extend to other annexins, including AnxA2, which together with S100A10, can bridge membranes (Illien et al., 2012; Grill et al., 2018; Berg Klenow et al., 2021), and bind to cholesterol-rich LE (Mayran et al., 2003). Similarly, AnxA8 is recruited to cholesterol-laden LE, and AnxA8 depletion caused LE/Lys cholesterol accumulation (Heitzig et al., 2018). Further examples of up- or downregulated tethers, with consequences for lipid- or ion-related MCS transfer, comprise phosphatase interacting protein 51 (PTPIP51, also called RMDN3) (Galmes et al., 2016), and PDZ domain-containing protein 8 (PDZD8) (Hirabayashi et al., 2017). Thus, manipulating the levels of tethers, untethers and lipid transporters (Galmes et al., 2016; Pulli et al., 2018) can offer therapeutic opportunities to modulate MCS formation in disease.

### Protein Domains in Annexins That Could Modulate MCS Assembly

The potential involvement of annexins in MCS formation by means of interactions with FFAT motifs (two phenylalanines in an acidic tract) in MCS-associated proteins should also be considered. FFAT motifs were originally identified in late endosomal/lysosomal proteins, interacting with ER-associated VAPs. Several variations of the original FFAT motif exist (Mikitova and Levine, 2012; Murphy and Levine, 2016; Cabukusta et al., 2020), including the Phospho-FFAT motif in AnxA5 and A8 (Di Mattia et al., 2020). Homologies to FFAT motifs were also located in AnxA1, A6 and A11 (Figure 1C) (Rentero et al., 2018). Given their association with the LE/Lys compartment and affinity for cholesterol (de Diego et al., 2002; Hulce et al., 2013), one could envisage MCS formation between the ER and LE/Lys being influenced by ER-associated VAPs or motile sperm domain-containing proteins (MOSPDs) recognizing FFAT-like motifs in these annexins.

Alternatively, post-translational modifications such as palmitoylation, which enables the targeting of cytosolic proteins to membranes, often modulating the activity of multiprotein complexes in specialized microdomains (Charollais and Van Der Goot, 2009). This is exemplified by palmitoylated caveolin-1 and its ability to bind cholesterol, thereby determining the cholesterol content of ER-mitochondria subdomains, linking organelle communication across MAMs with intracellular steroid and lipoprotein metabolism (Sala-Vila et al., 2016).

Likewise, palmitoylated cytoskeleton-associated protein 4 (CKAP4) interacts with VDAC2 at ER-mitochondrial contacts (Harada et al., 2020). This could impact on cholesterol transfer, as StARD1, which transfers ER-derived cholesterol to mitochondria, can form a complex with TOM22 and VDAC2 (see above) (Torres et al., 2017; Gordaliza-Alaguero et al., 2019).

Another example is the transmembrane protein 55B (TMEM55B), which interacts with the cytosolic scaffold protein JIP4 and dynein/dynactin in MCS to modulate the spatial distribution and positioning of lysosomes. TMEM55B palmitoylation was decisive for lysosomal positioning, implicating a critical role in determining the speed and location of MCS being formed, (Ballabio and Bonifacino, 2020; Rudnik et al., 2021; Saftig and Puertollano, 2021).

Interestingly, AnxA1 and AnxA6 were recently identified as palmitoylated substrates in extracellular vesicle fractions, including exosomes (Albacete-Albacete et al., 2020; Mariscal et al., 2020). The mechanisms that regulate this post-translational modification or whether this modification also applies for other annexins remains unknown. Hence, palmitoylated annexins may also contribute to MCS tethering. Exploring whether annexins establish palmitoylated links with perimeter LE/Lys membranes as well as mutational analysis of the FFAT-like motif in annexins will address the relevance and potential consequences of these proposed interactions.

### AnxA11 is a Tether of Lysosomes and RNA Granules

Recently, AnxA11 was detected in lysosomes that connect with RNA granules (Liao et al., 2019). Alike AnxA1 and A6, AnxA11 is widely expressed with diverse, often Ca^2+^- and S100A6 (calcyclin)-dependent functions in cytoplasmic and nuclear membrane locations, relevant for growth, cell cycle progression, differentiation, and exocytosis (Grewal et al., 2021).

Liao and coworkers identified AnxA11 as a tether mediating RNA granule association with lysosomes during their transport to distal regions of the axon (Liao et al., 2019). Strikingly, AnxA11 mutations associated with amyotrophic lateral sclerosis (ALS) disrupted docking between RNA granules and lysosomes, thus hampering neuronal RNA granule transport. Mutant analysis mapped the AnxA11 N-terminus as necessary for Ca^2+^- and phospholipid-dependent lysosome-RNA granule interactions, which could be relevant for RNA granule microtubule-based transport in polarized epithelial cells or neurons, facilitating local protein translation at subcellular locations (Lee et al., 2020; Das et al., 2021). Hence, AnxA11 represents a novel mechanistic and structural link between lysosomes and a membraneless compartment in ALS pathogenesis. This observation might extend to other annexins, as the AnxA11 interactome included AnxA7 (Liao et al., 2019). Similarly, using AnxA6 as bait, we identified AnxA11 and AnxA7 as binding partners, indicating that interactions between multiple annexins, as proposed previously (Li et al., 2016), may contribute to the tethering of lysosomes to other organelles.

## Discussion

Since the discovery of the annexin domain structure (Geisow, 1986), annexins have been identified in many organisms, including humans (Moss and Morgan, 2004). The Ca^2+^-inducible conformational change and differential preference for negatively charged phospholipids and other lipids, in particular cholesterol, can enhance membrane association of several annexins (Enrich et al., 2017). Furthermore, their promiscuous behaviour to differentially interact with other proteins together with their innate properties to “annex” membranes, make annexins suitable applicants for MCS appointments.

### Annexins: Regulators of Cholesterol Trafficking, ILV Formation and MCS Association

As outlined above, AnxA1 and AnxA6 control cholesterol transport from the ER to LE/Lys and vice versa *via* MCS (Eden et al., 2016; Meneses-Salas et al., 2020b). This contribution to MCS functioning may assist to control cholesterol levels in MVBs to participate, together with other lipids and accessory proteins (Gruenberg, 2020), in ILV biogenesis. This might even create transport specificity, as AnxA1 only mediates MCS formation between the ER and EGFR-containing MVBs (Wong L. H. et al., 2018).

However, annexin recruitment to MCS remains to be clarified. Besides the potential contribution of FFAT motifs or palmitoylation listed above, this could occur *via* translocation of cytosolic annexin pools where local Ca^2+^, cholesterol or annexin-binding proteins could contribute to their association with MCS. Alternatively, annexin pools inside ILVs could be released *via* back fusion, a constitutive process occurring in MVBs, where ILV fuse with the perimeter LE membrane leaving the cargo at cytosolic interfaces delimited by juxtaposed membranes of MVBs and other organelles (i.e., ER) (Gruenberg, 2020; Perrin et al., 2021). In this scenario, annexins could locally encounter a suitable Ca^2+^ and lipid microenvironment that would enable them to act as interorganelle tethers. On the other hand, annexins on ILVs facing the lumen of MVBs might be part of the fusion machinery (including LBPA, cholesterol, Alix) for the back fusion process*.* In fact, some of the complex protein networks that interact with AnxA6 are also involved in ILV formation and may regulate back fusion (Enrich et al., 2017). The well-documented presence of annexins in exosomes provides credibility for mechanisms such as chaperone-mediated autophagy (CMA) or endosome microautophagy (e-MI) for annexin association with ILV, the latter having the ability to keep proteins inside ILV. This is supported by all annexins harbouring the KFERQ motif (Figure 1C) (Cuervo et al., 2000; Kaushik and Cuervo, 2018), which is considered responsible for the location of annexins inside ILV (White et al., 2006; Meneses-Salas et al., 2020a).

Overall, the evidence of annexins contributing to MCS formation and function in LE/Lys is growing, with consequences for membrane traffic, microdomain organization, interactions with the cytoskeleton, cholesterol homeostasis, tethering, Ca^2+^ signalling and positioning of acidic compartments, and likely relevant for many biological settings.
